# Mycoremediation of lead and cadmium by lignocellulosic enzymes of *Pleurotus eryngii*

**DOI:** 10.1186/s13568-023-01626-8

**Published:** 2023-11-15

**Authors:** N. Goligar, S. Saadatmand, R. A. Khavarinejad

**Affiliations:** Department of Biology, Science and Research Branch, Islamic Azad University, Tehran, Iran

**Keywords:** Oyster mushrooms, *Pleurotus eryngii*, Cadmium, Lead, Mycoremediation, Sanger Sequencing

## Abstract

This study aimed to investigate the ability of *Pleurotus eryngii* fungus to absorb lead and cadmium from industrial wastewater. After culturing the fungus on a potato dextrose agar (PDA) medium containing 0 (control), 150 mg L^−1^, 250 mg L^−1^, and 350 mg L^−1^ concentrations of lead and cademium for 30 days, the mycelia were isolated from the culture medium and their extracts were used to measure protein content and the activity of antioxidant enzymes. Also, heavy metal contents were analyzed by atomic absorption spectrometry using flame photometry. Results showed that the growth of mycelia was significantly affected by different concentrations of the two heavy metals. High tolerance of heavy metal pollution in the culture media and the ability to accumulate lead and cademium confirmed that *Pleurotus eryngii* is a favorable option for mycoremediation. Also, molecular studies for fungal sequencing were investigated using the trench method, the sequence of the fungus was recorded in the gene bank, and finally the fungus was identified in the study.

## Key points

- Mycoremediation is a fast and low-cost heavy metal removal solutiok
- The proposed process does not add any harmful substances to the environment after cleaning.

## Introduction

Environmental pollution is a global problem. Due to enhanced industrial activities and rising living standards, today the environment is polluted by industrial wastes of various types (Singh et al. 2011). Environmental refining is defined as making use of organisms that occur naturally to eliminate or neutralize many non-toxic waste contaminants. Bioremediation is an environmentally friendly approach to recover contaminated environments (Azubuike Chikere and Okpokwasili, 2016). Within bioremediation approach, mycoremediation is performed by employing fungi that secrete intracellular and extracellular enzymes (Singh, 2010). This is the most complex type of bioremediation, in which mycelium is used to destroy polutants at the site of contamination (Thakur 2014). A number of fungal species have been reported to posses the ability to accumulate excessive environmental pollutants such as radionuclides (Bazala et al. 2005). In their study Bystrzejewska-Piotrowska et al. (2005) reported on the potential of *Pleurotus eryngii* mushroom to absorb cesium from the environment.

*Pleurotus eryngii* is an edible oyster mushroom with medicinal properties and applications in biotechnology and pharmaceutical industry (Cho et al. 2001; Gregori et al. 2007; Kang et al. 2000; Park and Jhune. 2010). This mushroom is commonly growing on dying trees, it behaves as a facultative parasite at the earliest opportunity. It lives on the roots and stems of living plants of the Apiaceae family (Zervakis et al. 2001; Lewinsohn et al. 2002), but from the viewpoint of forestry, it is primarily a saprophytic fungus speeding up wood decomposition. *Pleurotus eryngii* can be used for degradation of waste wood and is a white rot fungus. This mushroom can degraded phenolic compounds and waste wood due to having lignocellulosic enzymes. The lignocellulosic enzymes including lignin peroxidase (LiP), manganese peroxidase (MnP) and laccase responsible for degradation of lignin, celloluse and polycyclic aromatic hydrocarbons (PAH) and purify the pollution of the environment.

*Pleurotus eryngii* is developed in the Mediterranean, Central Europe, Central Asia, and North Africa. Among the 14 types of fungi, this mushroom has the highest concentration of natural estrogen and improves bone health (Shimizu et al. 2006). In addition, it reduces blood cholesterol (Alam et al. 2011) and is nutritionally considered as a rich source of proteins, carbohydrates, fibers, vitamins, and minerals (SyNytSyA et al. 2008; Krüzselyi et al. 2016; Caglarlrmak 2007; Wang et al. 2014; Zeng et al. 2012).

Fungi are equipped with antioxidant system that allows them to deal with heavy metals and other types of pollutants using a number of processes such as biological adsorption and biological conversion (Kulshreshtha Mathur and Bhatnagar, 2014). This ability is a property of their cell wall components and is dependent on the size and polarity of the fungal cell wall. In fact, the more metal binds to the functional groups of their cell wall, the higher the fungi ability to collect the metal (Dhankhar and Hooda 2011).

Over the past three decades, researchers have found that fungal species can contain heavy metal pollutants such as mercury, cadmium, lead, and metalloids such as arsenic and also radionuclides collected from the environment (Dogan et al.^2006^; Falandysz et al. 2008; García et al. 2015; Mendel et al. 2005; Uzun et al. 2011). In fact, removing pollutants from the environment through fungi is now seen as an economically viable solution (Ayangbenro 2017) with such advantages as the repeated use of biomass, selective metal bonding, effective adsorption, and recycling of the adsorbents. Fungi can chemically alter substances that affect their viability (Prakash 2017). The presence of chitin in their walls can help fungi tolerate high concentrations of metals and grow in an environment with low pH and temperature. Moreover, fungi and macrofungi have functional components that grow from a mycelial mass (Sarikurkcu et al. 2008; Synytsya et al. 2009). This improves their potential to be employed in mycoremediation to clean the environment from heavy metals and other pollutants. In this study, mycoremediation potential of *Pleurotus eryngii* oyster mushroom was investigated to remove lead and cadmium from industrial wastewaters through their lignocellulosic enzymes.

## Materials and method

### Culture conditions

*Pleurotus eryngii* strain IBRC-M 30529 with the sequence accession number MZ359864.1. *pleurotus erynjii* was cultured in a potato dextrose agar (PDA) medium. Then, heavy metals Cd and Pb were added to the culture at concentrations of 0, 150, 250, and 350 mg L^−1^. After 30 days, the mycelia were removed from the culture media, and the effects of the heavy metals on the treatment and control group were investigated.

### Heavy metal assay

Concentration of microelements (lead and cademium heavy metals) absorbed by the *Pleurotus eryngii* during the treatment period was assayed spectrometrically following the method described in Bempah et al. (2013). The equipment and devices were first washed with HNO_3_ 3% solution; then, the fungus mycelia were removed by using gloves, and after adding nitric acid, the mycelia kept at laboratory temperature for 24 h for complete acid digestion. The obtained extracts were then passed through a 10 mm filter paper and transferred to test tubes by a vacuum pump. Flame atomic absorbtion method was use to measure the concentration of heavy metals in terms of mgg^−1^ of dry weight with an atomic absorption spectrophotometer (Shimadzu AAS 80).

### Soluble protein assay

To measure soluble protein contents of fungal material, Bradford protein assay (1976) was used. This is a colorimetric method for measuring the concentration of a protein based on forming a complex of the dye Coomassie Brilliant Blue G-250 with the proteins available in a solution. When the dye, binds to the protein, it causes a shift from 465 to 595 nm, which is why the absorbance is read at 595 nm.

### Superoxide dismutase (SOD) activity assay

SOD enzyme activity assay was performed using the method of Giannopolitis et al. (1977). The enzymatic reaction mixture consisted of 935 μl of phosphate buffer 50 mM containing EDTA 0.1 mM, methionine 13 mM, Nitro blue tetrazolium (NBT) 75 μm, 15 μl of riboflavin 0.12 mM, and 50 μl of enzyme extract. After preparing the control and blank samples, the blank sample was in the dark for 15 min, and the control samples together with enzyme extract were shaken at 100 rpm for 15 min in a shaker with two 20 w fluorescent lamps at 25 ℃. Then, the absorption was read at a wavelength of 560 nm by the spectrophotometer. Finally, the activity of the SOD enzyme was calculated using the following formula, and expressed as μmg^−1^ (Giannopolitis et al. (1977):

$$SODactivity\left( {{\raise0.7ex\hbox{${Unit}$} \!\mathord{\left/ {\vphantom {{Unit} {gFW}}}\right.\kern-0pt} \!\lower0.7ex\hbox{${gFW}$}}} \right) = \frac{{100 - \left[ {\frac{{\left( {ODcontrol - ODsample} \right)}}{ODcontrol} \times 100} \right]}}{50}$$

where FW denotes fresh weight, and OD Control and OD sample are optical dencities for control and samples, respectively.

### Phenolic assay

In order to measure phenolic contents of the fungus mycelia, 0.5 ml of the fungal extract was mixed with 0.5 ml of Folin-Ciocalteu reagent solution and 0.05 ml of sodium carbonate solution. The absorbance was read at 760 nm after 1 h of stirring in against the blank. Gallic acid was used as the standard for drawing the calibration curve. The experiments were repeated three times, and their average values were reported (Atoui et al. 2005; Ordonez et al. 2006).

### Flavonoid assay

Methanol was used for extraction through percolation method. The solvent evaporated in a vacuum, and the collection was dried by a freez dryer (Fathi et al. 2011). An aluminum chloride reagent was used to measure the amount of flavonoid. Methanol, 10% aluminum chloride solution in ethanol, 1 m of potassium acetate solution, and distilled water were added to the fungi extract. The absorption of the mixture was read half an hour later with a spectrophotometer at 420 nm against the blank. Quercetin was used as the standard for drawing the calibration curve.

### DPPH assay

Different volumes of the extract (150–350 μl) was increased to 3 ml by adding absolute methanol. Then, 1 ml of the solution 0.004% DPPH in methanol was added and mixed well. After 30 min at room temperature in the dark, the light absorption of the samples was read at 517 nm wavelength. The percentage of scavenging free radicals by DPPH was calculated by the following formula (Akowuah et al. 2005):

$$I\% = [(A_{blank} - A_{sample} )/A_{blank} )] \times 100$$

where I, A_blank_, and A_sample_ are the free radical scavenging capacity (%), light absorption of thenegative control (without extract), and light absorption of the samples with various concentrations of the extract, respectively. The synthetic antioxidant butylated hydroxytoluene (BHT) was used as a positive control.

### Peroxidase activity assay

The supernatant of the fungal extract solution was used for peroxidase enzyme assay. To measure peroxidase activity, 3 ml of a mixture consisting of 1950 mM potassium phosphate buffer (pH 6.1), 500 mM guaiacol, and 500 mM hydrogen peroxide (H_2_O_2_) was prepared. Then, the mixture was added to 50 μmol of the enzymatic extract, and its optical density was measured at 470 nm for 1 min (Kjalke et al. 1992).

### Lignin peroxidase activity assay

To measure the activity of peroxidase enzyme, 1 ml of a 50 mM potassium sodium tartrate buffer (pH 4) and 0.1 ml of 0.1 mM H_2_O_2_ were added to 10 ml of the solution containing the enzyme, and the enzyme activity measurement was performed at a wavelength of 650 nm (Kjalke et al. 1992).

### Laccase activity assay

To measure the activity of laccase, the fungal samples were ground and centrifuged in order to extract laccase in 100 mM of sodium acetate buffer (pH 5) at 4 ℃. Then, 0.1 ml of a 50 ml potassium sodium tartrate buffer (pH 3), and 0.4 ml of veratrine alcohol were added to 1.65 ml of the extract to obtain 3 ml mixture. Next, 0.2 mM of H_2_O_2_ was added, and the enzyme activity was read at 310 nm (Archibald and Roy 1992). The enzyme activity was read by a spectrophotometer in mg/protein as a change in absorption at 420 nm after1 min (Odusanya 2003; Safari et al. 2006).

### Statistical analysis

In this study, all the analyses were repeated three times. The data was analyzed, and the graphs were drawn with GraphPad 8. Analysis of variance (ANOVA) of the data was performed, and the means of the recorded values were compared using Duncan's test (P ≤ 0.001).

## Results

**Fig. 1:**
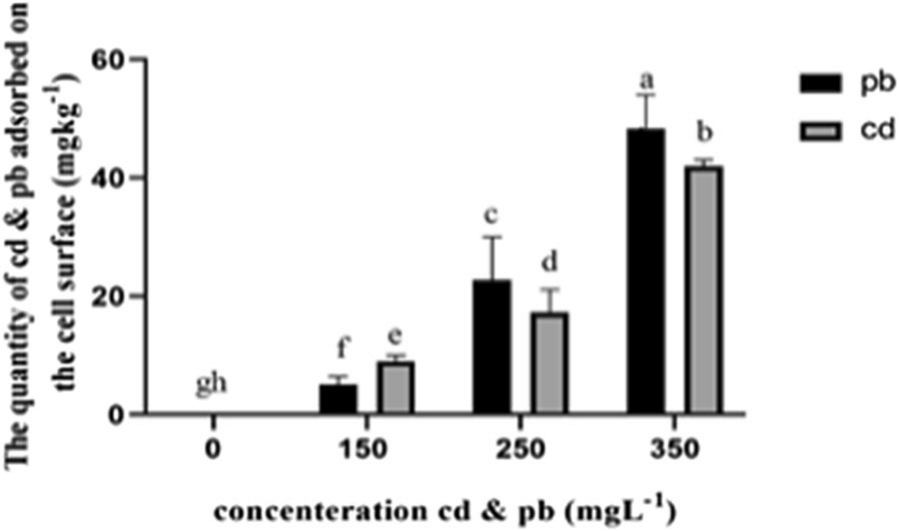
The amount of lead adsorbed by the mycelial surface of *Pleurotus eryngii*. The mycelia were collected and washed with ddH_2_O to remove the residues of the medium, and then washed with 150 ml 0.1 M EDTA for 60 min, with shaking at 100 rpm. After that, the mycelia were rinsed with ddH_2_O. Pb and Cd adsorbed onto the cell surface was measured in both solutions. All the values are the mean of three different samples ± standard error. The bars for each metal marked by different letters are statistically different at *p* < 0.01

Results of ANOVA also showed that various concentrations of heavy metals significantly affected cademium and lead accumulation by the fungi in the present study (Fig. 1).

In the fungi treated with the culture medium containing 350 mg L^−1^, mean accumulation of lead (48.8 mg^−1^) was significantly higher in comparison with the control and other treatments (P ≤ 0.001). The analyses of variance of the obtained data revealed that increasing concentrations of cadmium and lead in the culture (from 150 to 350 mg L^−1^) affected accumulation of these pollutants by fungal mycelia, significantly increasing their removal in comparison with the control group. Also, the absorption of lead was generally higher than cadmium (Fig. 1).

**Fig. 2:**
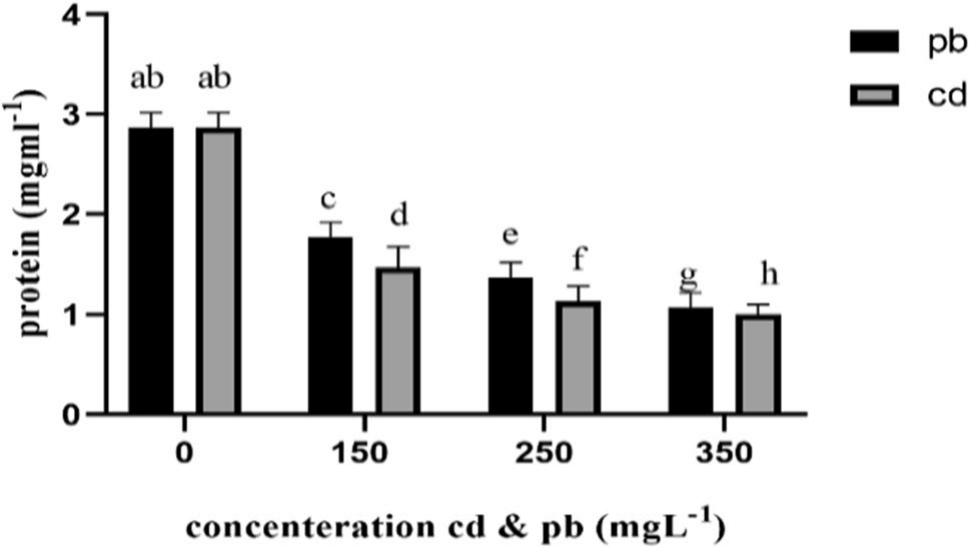
Bar Chart showing protein content of *Pleurotus eryngii* fungi under the influence of heavy metals. Bradford protein assay used to measure protein concentration in solution. The highest amount of protein was observed in the control mushroom

In addition, protein contents of the fungal mycelia decreased with increasing concentration of cadmium and lead in the media culture from 150 to 350 mg L^−1^, showing significant differences from the control group (Fig. 2).

**Fig. 3:**
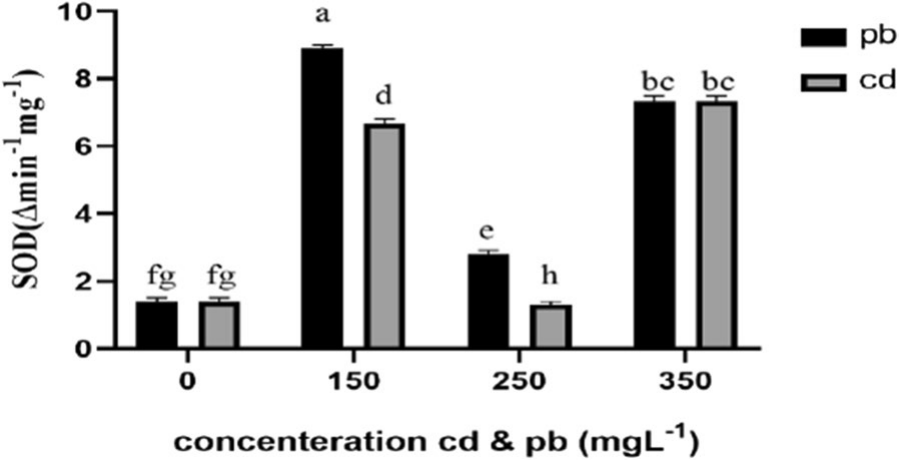
Effect of heavy metal concentration on activities of superoxide dismutase (SOD) in *Pleurotus eryngii*. SOD activity was determined according to Giannopolitis et al. method (1997). The highest amount of SOD enzyme was observed in mushrooms treated with 150 mgL^−1^ concentration

**Fig. 4:**
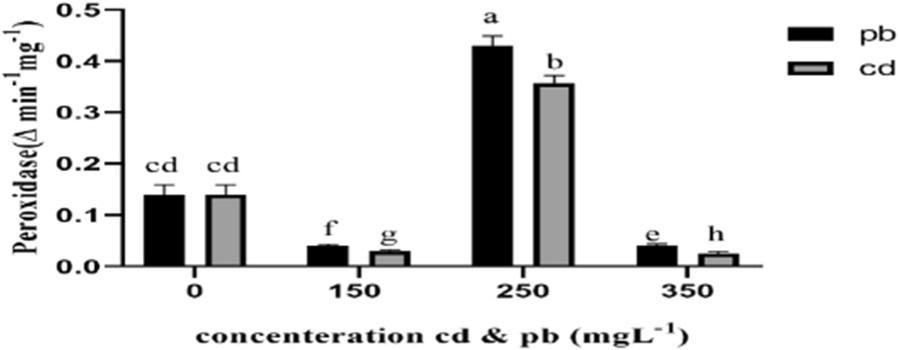
Peroxidase activities were determined according to the method of Kjalke et al. (1992). The highest amount of peroxidase in 250 mgL^−1^ pb and cd treatments was observed. The treatments lower and higher than this value showed a significant decrease in the amount of peroxidase activity compared to the control sample

**Fig. 5:**
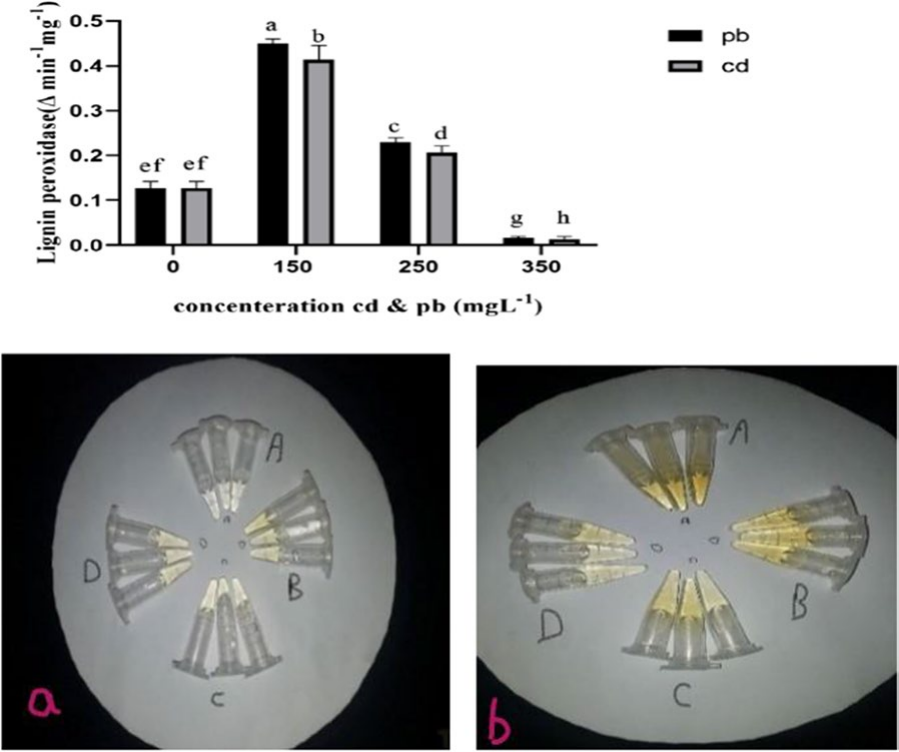
Lignin peroxidase activities were determined according to the method Kjalke et al. 1992. Optimum lignin peroxidase activities were observed at a concentration of 150 mgL^−1^. Figure 8.a. **A**: (0) Control, **B**:150, **C**:250, **D**: 350 mgL^−1^ Lead. b. **A**: (0) Control, **B**:150,**C**:250, **D** 350 mgL^−1^ cadmium. Shapes a and b show that in the higher concentration of lead and cadmium, the brighter the color of the extract

**Fig. 6:**
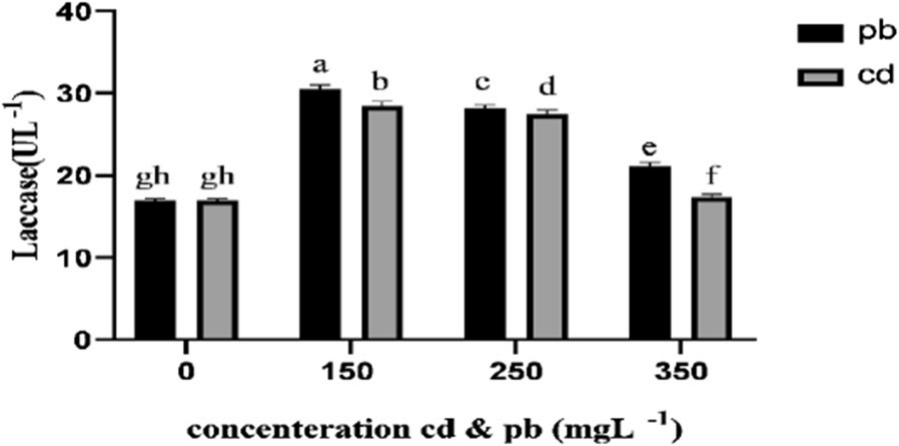
Laccase enzyme activities was performed according to Odusanya (2003); Safari et al. (2006). The highest amount of laccasc activity was observed in mushrooms treated with 150 mgL^−1^ cd and pb

Antioxidant enzyme contents of the mycelia generally changed in *Pleurotus eryngii* fungi exposed to various concentrations of lead and cadmium (Figs. 3, 4, 5). The treatment containing 150 L^−1^ lead resulted in the maximum increase in superoxide dismutase content of fungal mycelium, which was significantly different from the control group. This was followed by the treatment with cadmium 150 L^−1^, which showed a significant differnece from the control fungi (Fig. 3). Results of ANOVA showed that peroxidase and lignin peroxidase enzyme contents of the mycelium in the fungi treated with 250 and 150 mg L^−1^ lead and cadmium, increased significantly in comparison with the control group (Figs. 4, 5). On the other hand, the higher concentration of these heavy metals (350 mg L^−1^) decreased the activity of these enzymes. Findings also showed that cadmium and lead significantly increased the activity of laccase enzyme compared to the control group (Fig. 6).

**Fig. 7:**
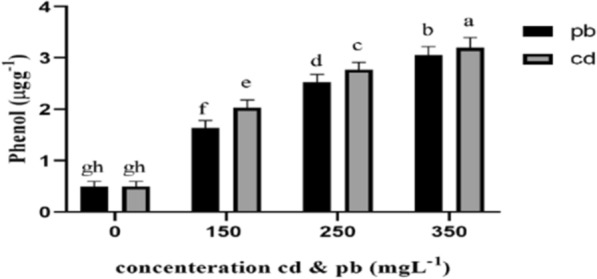
Total phenolic content under the influence of heavy metals (cd and pb). The highest amount of phenol was observed in the treatment 350 mgL^−1^ cd and pb. With the increase of lead and cadmium concentration, the amount of phenolic compounds increased significantly

**Fig. 8:**
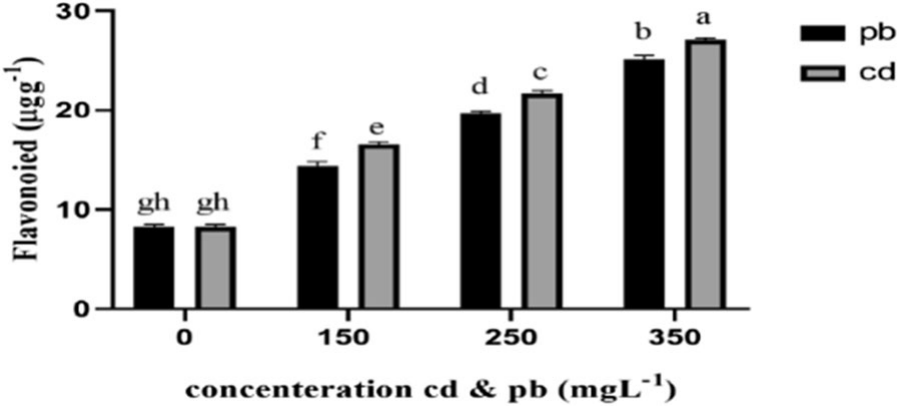
The content of flavonoids in *Pleurotus eryngi*i mycelia. The highest amount of flavonoids was observed in mushrooms treated with 350 mgL^−1^ cd and pb

**Fig. 9:**
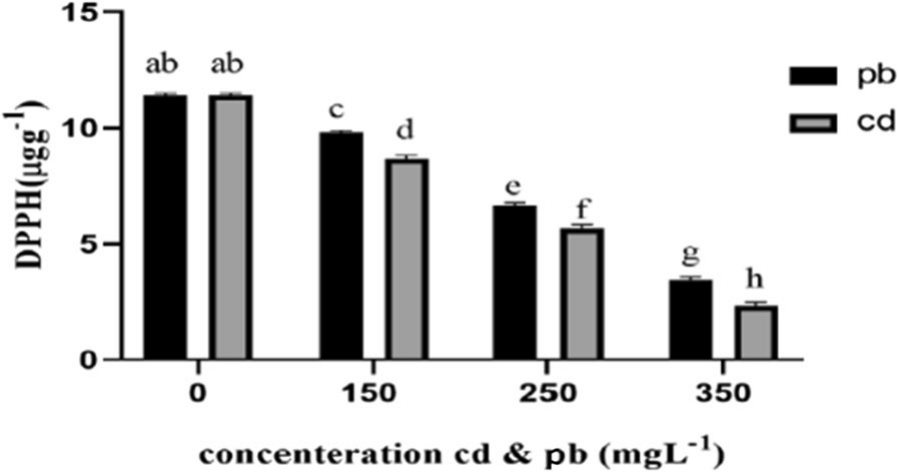
DPPH scavenging activity in concentrations of 0 (control), 150, 250 and 350 mgL^−1^ Pb and Cd. The highest amount of DPPH in the control treatment was observed

Finally, results of ANOVA tables showed that increase in cadmium and lead concentrations from 150 to 350 mg L^−1^ significantly affected phenols, flavonoids, and DPPH contents of the fungal mycelia, significantly increasing the level of these compounds compared to the control. The increase in phenolic compounds under cadmium stress was more than that of lead treatments (Figs. 7, 8, and 9).

## Discussion

Basidiomycetes, including *P. eryngii* absorb heavy metals through mycelia. In the absorption mechanism, known as passive uptake, physical absorption or ion exchange occur at cell surface, reaching the adsorption equilibrum within 30–40 min (Das et al. 2008). There are two separate passive ion transfer paths in the mycelium of fungi. In intracellular path, ions transfer via hyphae surrounded by septa while extracellular path is concerned with the transfer via the cavities between the hyphae and the matrix.

Findings of the present study showed an increase in the accumulation of lead and cademium by fungal mycelia with increasing concentrations of cadmium and lead in the culture (from 150 to 350 mg L^−1^). This is in line with the findings of Dawn et al. (2006) who also reported the accumulation of lead (15.2 mg/kg) and cadmium (0.31 mg/kg) in the fruiting body of *P. eryngii* mushroom.

A number of factors affect the concentration of accumulated heavy metals. Heavy metal accumulation is primarily dependent on the fungus species. The absorption capacity of the mycelia might be a function of overall surface area of the fungal hyphae. It is also influenced by the acidic and organic substances of the ecosystem and soil (Gast et al. 1988). Moreover, biosorption was argued to decrease with an increase in biosorbent particle size and its concentration (Zhou 1999). Fourest and Roux (1992) attributed the decrease in specific uptake might to low metal concentration in the solution. Also, biomass concentration in the solution seems to influence heavy metal uptake by the fungi. For lower biomass values, high concentration of heavy metals results in an interference between the binding sites (Das et al. 2008).

Protein contents of the fungi mycelia in the control group (2.86) was significantly higher than the other treatments of the study (P ≤ 0.001). Nutrient depletion, e.g. that of fungal protein under heavy metal treatments has been reported earlier in the literature. For example, signs of reduced mycelial protein contents were robserved in *A. bisporus* and *p.ostreatus* due to the accumulation of mercury, cadmium, lead, and zinc (Lasota et al. 1990).

Results of ANOVA showed a general change in antioxidant enzyme activities of the mycelia in the fungi treated with various concentrations of lead and cadmium.Due to the disruption of cell homeostasis, heavy metals pose abiotic stresses in plants, causing an increase in hydrogen peroxide levels in them (Sharma et al. 2005). Like other plants, algae adopt enzymatic and non-enzymatic antioxidant defence mechanisms to scavange reactive oxygen specied (ROS) in an attempt to mitigate the adverse effects of the biotic or abiotic stresses. Increased level of ROS and other free radicals upregulates the activity of the antioxidant enzymes and non-enzymes in plants deponding on the intensity, duration, and type of stress (Sharma et al. 2012).

A significant increase in SOD activity was observed in *Pleurotus eryngii* fungi under treated with heavy metal stress compared to the control. Superoxide dismutase is the sole enzyme of the antioxidative defense system which eliminates superoxide by catalyzing the destruction of superoxide anion (O_2_^−^) into H_2_O_2_ and elemental oxygen (O_2_) (Shi et al. 2007). In most studies, the direction of SOD activity is reported as variable which is partly due to differences in experimental factors such as fungi species, tissue type, metal type, metal concentration, and duration of metal exposure (Chin 2007); Mirsha et al. (2006) exposed *Ceratophyllum demersum* plants to lead (10–100 μm) for a period of 2 to 7 days, and found that the activity of SOD enzyme increased at low concentrations and with a short period of exposure. Sharma et al. (2012) also reported a decrease in SOD activity with increasing concentration and duration of the heavy metal stress.

While lower levels of lead and cadmium (150 and 250 mg L^−1^) significantly increased peroxidase and lignin peroxidase enzyme contents of the mycelium in the fungi under study in comparison with the control group, a drop in the activities of these enzymes was observed under 350 mg L^−1^ heavy metal treatment. Peroxidase plays a pivotal role in combating oxidative damages inflicted by heavy metals in plants (Verma et al. 2003). At high concentrations of heavy metals and upon long exposure, the level of enzyme activity decreases owing to an increase in ROS production, disruption of cell metabolism, and the induction of oxidative stress (Kaur et al. 2012). Low peroxidase activity observed in the fungi treated with higher concentrations of heavy metal might be due to disrupted membrane permeability and cell damage, a common effect of free radicals (Bhardwaj et al. 2001).

Cadmium and lead significantly increased laccase enzyme levels in comparison with the control. Laccase belongs to a group of polyphenol oxidases containing copper atoms on the catalytic site and is commonly considered as a multiple oxidase with a relatively low redox potential (0.8–0.4) and (Palmer et al. 1999). Laccases are extracellular glycoproteins that catalyze the organic and mineral oxidation of a broad number of phenolic and non-phenolic substrates and Mn^2+^ by using molecular oxygen as the electron receptor, which is reduced to water while Cu^+2^ is reduced to Cu^+^ (Munoz et al.^1997^; Thurston 1994). Cu^+2^ participates in the regulation of laccase gene transcription (Galhaup and Haltrich 2001; Palmeiri et al. 2000) and has positive effects on activity and stability of this enzyme (Baldrian et al. 2002). Ordonez et al. (2006) reported that Cu^+2^ (1 mM) reduced the activity of extracellular protease, which is responsible for the destruction of laccase. On the other hand, Munoz et al. 1997 reported that CuSO_4_ at 5 mM concentration did not affect laccase activity. Stajic et al. (2006) recorded the highest level of laccase activity under 1 mM concentration of Cu^+2^, which showed a significant decrease under higher concentrations of Cu^+2^. Since the susceptibility of the fungi to Cu^+2^ and Cd^+2^ changes over time, laccase induction occurs only when cadmium is added in later stages of growth (Baldrian et al. 2002).

It was found in the present study that increasing the concentration of cadmium and lead from 150 to 350 mg L^−1^ improved the phenols, flavonoids, and DPPH contents of mycelia, showing a significant difference from the control. These antioxidants trap the released free radicals that cause toxicity in plants (Alinejad et al. 2016). Phenolic compounds in the mycelia of *Pleurotus eryngii* fungus increased under heavy metal treatments in this study. These are significant secondary metabolites formed in response to environmental stresses. These metabolites are partially responsible for the strong antioxidant properties in fungi, as they generally help prevent cell damage caused by free radicals (Ryan-Harshman and Aldoori 2005). The capability of phenolic compounds to clean free radicals is believed to depend on the number of aromatic rings and the nature of the moving hydroxyl groups (Lagouri and Boskou 1996). Hydroxyl groups (OH^−^) in these compounds can neutralize free radicals and act as electron or hydrogen transmitters (Fukumoto and Mazza 2000; Pandey and Tripathi 2011). Finally, DPPH is a stable free radical with a nitrogen atom at the center, whose color changes from purple to yellow by oxidation processes. By providing an electron or hydrogen atom, antioxidants or other radical species can react with DPPH and reduce it to 2,2-diphenyl-1-hydrazine (DPPH-H) or a substituted analogous hydrazine (DPPH-R).

Findings of the study showed that *P. eryngii* can expunge heavy metals from the substrate environment with high efficiency. Biosorption of heavy metals by fungi through mycoremediation seems to be a an environmentally friendly, convenient, and inexpensive approach to eliminate heavy metal contaminants from soil and water resources. However, accumulation of excess heavy metals can in return adversely affect bioacumulating alage morphophysiologically. Various factors must be considered for optimal redemption of heavy metals from the environment. Investigation of the efficacy of heavy metal uptake by microbial biomass is essential for industrial wastewater treatment (Ahalya et al. 2003). In fact, the search for appropriate species of fungi to accumulate heavy metal pollutants from the environment and optimal conditions for biosorption is an ongoing process. This paper presented the findings of a small-scale laboratory experiment, which might be considered as preliminary experimental results. It is suggested to conduct new studies to probe into the mechanisms of physiological and biochemical absorption by fungai species under more natural conditions.
